# Smaller Radius Width in Women With Distal Radius Fractures Compared to Women Without Fractures

**DOI:** 10.7759/cureus.1950

**Published:** 2017-12-14

**Authors:** Gary Kiebzak, Walter R Sassard

**Affiliations:** 1 Orthopedic Surgery, Nemours Children's Hospital; 2 Orthopaedic Surgery, Houston Methodist Hospital

**Keywords:** radius fracture, bone width, dual-energy x-ray absorptiometry

## Abstract

Introduction

Bone mineral density (BMD) measured using dual-energy x-ray absorptiometry (DXA) is typically used to assess fracture risk. However, other factors such as bone size and the forward momentum of a fall (a function of body size) can also potentially influence fracture risk, but are understudied. This report describes the characteristics of a cohort of Caucasian pre- and postmenopausal women with distal radius fractures (DRF) after falling onto an outstretched hand.

Methods

The fracture cohort comprised entries in an institutional review board-approved registry of study patients who had had DXA scans. For patients with DRF, the contralateral radius was scanned and BMD, T-scores (used to define bone status as normal, osteopenic, or osteoporotic), and radius width were recorded. Generally, side-to-side (left-right) differences in bone size and BMD are small and, hence, the contralateral radius was considered a surrogate for bone status of the fractured radius. Apparently healthy women without fractures were used as race-, age-, and BMI-matched controls.

Results

Premenopausal women < 49 years of age (mean age, 38 years) with DRF had significanty smaller radii width compared to matched controls. Mean radius BMD was in the normal range. As a group, the cohort was overweight based on mean BMI. Postmenopausal women > 50 years (mean age, 64 years) with DRF also had low radius width, but in contrast to the first group, this group had low peripheral and central BMD.

Conclusions

Women with DRF had contralateral and presumably fractured radii of bone width smaller than matched controls. As a group, these women were also overweight based on BMI. The smaller radius width may increase the risk for fracture irrespective of BMD, especially since larger body size would result in greater inertial force when falling while ambulating.

## Introduction

Early studies with dual-energy x-ray absorptiometry (DXA) helped instill the prevailing concept that low energy ("atraumatic") distal radius fractures (DRF) in postmenopausal women are related to low bone mineral density (BMD) [1-3].  A typical event resulting in this type of fracture is when a patient falls forward and attempts to break the fall with an outstretched arm [1-4].  BMD is commonly considered to be indicative of bone strength. This is because low BMD is associated with either smaller bone size or bones characterized by negative structural changes such as microarchitectural deterioration, thinning cortices, endosteal porosities, etc. [5-8]. If BMD is low, then the impact force upon falling onto an outstretched arm can exceed the mean strength of the distal radius leading to a fracture [7-9]. The incidence of DRFs increases in the early-to-mid 50s for women and, chronologically, these fractures represent the first type of fracture related to osteoporosis (followed by vertebral and hip fractures) [10].

However, it is important to recognize that other factors contribute to the risk of DRF in addition to BMD. It is well established that bone size is highly correlated to the ability of the bone to resist deformation and, ultimately, fracture. In fact, one of the reasons why BMD is a good surrogate of biomechanical measures of bone strength is that BMD is in part a reflection of bone size when measured by DXA, because DXA does not totally correct for bone size (bone mineral content (BMC) is an even better reflection of bone size than BMD). Bone size is expressed by various parameters such as bone area, width of the cortices, or even width of the whole bone at the region of interest, which could be used as an estimate of cross-sectional area.  

Other factors that, at least theoretically, influence risk for DRF include body mass, height, and speed of movement at the moment of impact from a fall. That is, the linear momentum (p) of a body is the product of its mass (m) and its velocity (v), i.e., p = mv. High forward momentum would increase the impact force generated upon a fall. However, these factors have not been thoroughly studied with respect to the mechanism of DRF across all age groups.

Over the course of seven years, DXA results from several unrelated studies were entered into an ongoing IRB-approved database. The objective of this study was to summarize and compare demographic and DXA data between women with DRFs, and race-, age-, and BMI-matched women without fractures.

## Materials and methods

Studies and scans were done at the Center for Orthopedic Research and Education, St. Luke's Episcopal Hospital, Houston, TX. Patients who volunteered and signed an informed consent form to participate in one of several institutional review board-approved studies involving a DXA scan were added to the DXA database. Basic demographic data were entered, and brief histories were taken to record menopause status and current or prior use of medications that affect bone (anabolics, antiresorptives, etc.).

A total of 120 patients had a DXA scan within three months of the fracture. Fractures were classified as either unstable and/or displaced fractures, and either as intraarticular or extraarticular. All types of radius fractures were included. Concomitant ulna fracture was not exclusionary.

For this study, inclusion criteria were: Caucasian (86% of fracture patients were Caucasian, and this eliminated possible bias due to known race differences with BMD), and having a low-energy fracture. DRF were considered to be low energy if they resulted from a fall from a standing height while moving forward (as in walking), or if they resulted from a fall from a stationary position such as fall from a low height, such as a one- or two-step household stepstool. Most patients sustained the DRF while tripping or slipping while walking, and in characteristic fashion, tried to break the fall with an outstretched arm. Exclusion criteria were: a DRF from a high-energy event such as a motor vehicle accident, a fracture of any kind of the scanned contralateral limb, history of metabolic conditions affecting the bone, or having started medications known to affect bone metabolism (antiresorptives or anabolics) at the time of fracture. The resultant sample size was 98 cases. Sixty-two percent of the fractures were on the nondominant side.

There were 355 women in the DXA database without DRF. Of these, 102 had DXA scans of both forearms. 

Preliminary analyses showed various significant relationships between age, BMI, radius BMD of the DXA 33% region of interest (ROI), and width in the control cohort (no fractures, n = 355). For example, BMI was significantly correlated with BMD of 33% ROI (r = 0.71, P < 0.0001), and width of the 33% ROI (r = 0.324, P < 0.0001). Age and BMD of the 33% ROI were significantly correlated ( r = -0.25, P < 0.0001). Width and BMD of the 33% ROI were significantly correlated (r = 0.227, P < 0.00001). Width increased as a function of BMI class for underweight (1.36 + 0.27 cm), normal weight (1.33 + 0.21 cm), overweight (1.41 + 0.25 cm), and obese (1.52 + 0.27 cm) patients. Consequently, to have a comparable group without fractures, it was necessary to adjust for age and BMI. Patients without DRF were matched to the patients with DRF in the following manner. First, only Caucasian women were selected. Of these, patients with borderline ages were eliminated until the mean age equaled that of the DRF patient cohort. Of these, the same maneuver was done for BMI until the mean BMI was almost equal to that of the DRF patient cohort.

Considering the possible effect of dominant side on bone width and BMD (and resultant T-scores), preliminary analyses were done comparing patients with dominant side fractures to nondominant side fractures; bone width and BMD of the contralateral radius were not significantly different (data not shown). The same was done for the larger group of patients without fractures who had both forearms scanned (n = 102); the differences between dominant and nondominant radii were significant but small, 1.1% for width (P < 0.034) and 2.2 % for BMD (P< 0.003) respectively (calculated using the paired t-test), and considered clinically irrelevant. Thus, data for dominant and nondominant sides were pooled for both DRF patients and matched controls.

All patients were scanned using standard methods on a calibrated GE Lunar Prodigy DXA system (using Encore software; General Electric Company, New York) that was dedicated for research studies. All patients with DRF had a single measurement of the contralateral nonfractured forearm, lumbar spine, and proximal femur. Regions of interest (ROIs) were: ultradistal radius (UDR), 33% (a region adjacent to one-third the length of the ulna indexed at the tip of the ulnar styloid process), total radius, L1-4, femoral neck, and bilateral total hip. All scans were reviewed and interpreted by the same investigator. Patient bone status was determined using WHO criteria. If normal, all T-scores from all ROIs were > -1.0. The lowest T-score from any ROI except the ultradistal radius (UDR) was used to categorize bone status as osteopenic or osteoporotic. In addition to BMD and T-scores, the width of the radius at the 33% ROI was obtained by dividing the 2 x 2 cm ROI area by 2.0; there was no ruler tool available to directly measure width. The width of the ultradistal ROI could not be reproducibly obtained due to the irregular shape of this ROI (in contrast to the 33% ROI which is always a rectangular or square shape, and the midpoint easily measured).

Women were divided by menopausal status (pre and post) at the time of fracture which, for this cohort, resulted in an age cutoff point of 49 years old and younger and 50 years old and older. Comparisons between groups were made using the unpaired t-test, with significance at P < 0.05 (using Instat2, Graphpad Software, San Diego). 

## Results

Table 1 summarizes the demographic data for the patients with DRF and controls. For premenopausal women, the age range was 20-49 years, for postmenopausal women, 50-80 years. Weight and height were greater in premenopausal women with DRF than postmenopausal women with DRF. 

**Table 1:** Demographics for patient groups Values are mean + SD. NS: not statistically significantly different. * Controls were Caucasian women with no fractures not taking bone-related medications from a database of apparently healthy study volunteers.

	Age, yrs	Height, in	Weight, lbs	BMI
Caucasian women with distal radius fracture				
Premenopausal, <49 yrs, n = 36	38 + 10	65.9 + 2.5	172 + 46.7	27.6 + 6.5
Postmenopausal, >50 yrs, n = 62	64 + 9.9	64.1 + 3.0	154 + 29.4	26.4 + 5.2
Comparing pre- to postmenopausal, P	< 0.0001	0.003	0.021	NS
Controls matched for race, age, BMI*				
Premenopausal, <49 yrs, n = 65	38 + 9.7	65.2 + 2.6	170 + 29.5	28.0 + 4.8
Postmenopausal, >50 yrs, n = 100	64 + 8.0	64.2 + 2.7	155 + 33.7	26.8 + 5.3
Comparing pre- to postmenopausal, P	<0.0001	0.020	0.004	NS

Mean T-scores for the premenopausal women were in the normal range as defined by WHO criteria; 66.7% were normal, and 33.3% were osteopenic based on the lowest T-score from either 33% radius, femoral neck, total hip, or lumbar spine. In contrast but as expected, mean T-scores for older postmenopausal women verified low BMD (Table 2); 11.3% were normal, 50.0% were osteopenic, and 38.7% osteoporotic. T-scores for premenopasual fracture patients were similar to age-, weight-, height-, and BMI-matched controls. T-scores for postmenopausal fracture patients were significantly lower than those for matched controls.

**Table 2:** T-scores for patient groups 1 P < 0.029 compared to matched normal controls, unpaired t-test. 2 P < 0.01 (or less) compared to matched controls > 50 years of age. Values are mean + SD. DXA regions of interest are as follows: UDR, ultradistal radius; 33%, approximately one third radius; FN, femoral neck; L1-4, lumbar vertebral bodies 1-4. * Controls were Caucasian women with no fractures not taking bone-related medications from a database of apparently healthy study volunteers.

	Radius			Hip		Spine
Caucasian women with distal radius fracture	UDR	33%	Total	FN	Total	L1-4
Premenopausal, <49 yrs, n = 36	-0.8 + 1.3^1^	-0.2 + 0.9	-0.3 + 1.1	-0.2 + 1.1	-0.1 + 1.3	-0.1 + 1.0
Postmenopausal, >50 yrs, n = 62	-2.2 + 1.4^2^	-1.6 + 1.2^2^	-1.8 + 1.4^2^	-1.7 + 0.8^2^	-1.4 + 0.8^2^	-1.6 + 1.4^2^
Comparing pre- to postmenopausal, P	<0.0001	<0.0001	<0.0001	<0.0001	<0.0001	<0.0001
Controls matched for race, age, BMI*						
Premenopausal, <49 yrs, n = 65	-0.2 + 1.3	-0.1 + 0.8	-0.1 + 1.1	-0.1 + 1.2	0.1 + 1.1	0.4 + 1.2
Postmenopausal, >50 yrs, n = 100	-1.5 + 1.7	-1.0 + 1.2	-1.2 + 1.5	-1.1 + 1.0	-0.8 + 1.6	-0.8 + 1.7
Comparing pre- to postmenopausal, P	<0.0001	<0.0001	<0.0001	<0.0001	0.029	0.001

Bone width at the 33% radius ROI was statistically significantly smaller (16%) for premenopausal fracture patients compared to matched controls (Table 3). The difference between postmenopausal fracture patients and controls was less (8%), but still significant.

**Table 3:** Width of radius at 33% ROI Values are mean + SD. NS, not statistically significantly different. 1 P < 0.0001 compared to race-, age-, BMI-matched controls. 2 P < 0.001 compared to race-, age-, BMI-matched controls.

	Width, cm
Caucasian women with distal radius fracture	
Premenopausal <49 yrs, n = 36	1.22 + 0.11^1^
Postmenopausal >50 yrs, n = 62	1.26 + 0.14^2^
Comparing pre- to postmenopausal	NS
Controls matched for race, age, BMI	
Premenopausal, <49 yrs, n = 65	1.45 + 0.25
Postmenopausal, >50 yrs, n = 100	1.37 + 0.24
Comparing pre- to postmenopausal, P	0.041

All but two of the DRF were unstable, and 84% were displaced. For both the pre- and postmenopausal women, 48% were extraarticular and 52% intraarticular. The percentage of intraarticular fractures (most severe) increased with increasing BMI; for BMI of < 25, > 25, and > 30, the percent of patients with intraarticular fractures was 32%, 55%, and 60% respectively.

## Discussion

Women with DRF had contralateral radii characterized by small bone width relative to body size (as defined by BMI), when compared to race-, age-, and BMI-matched women without fractures. Smaller bone width equates to smaller cross-sectional area and may increase the risk for fracture irrespective of BMD, as premenopausal women (< 49 years) had mean T-scores in the normal range. The mean BMI for both pre- and postmenopasual women was in the overweight category, but younger premenopausal women were taller with greater body weight than the older postmenopausal women with fractures. In addition, T-scores were lower in postmenopausal women. Thus, there were differences not only between the cohort with DRF and controls, but also within the DRF cohort. Therefore, the circumstantial setting of fracture may be different for each group. 

In postmenopausal women, the circumstantial setting of fracture is probably related mainly to the low BMD characteristic of older women with DRF. In contrast, premenopausal women had normal BMD. (Normal BMD is not necessarily surprising as this group was much younger; mean age was 38 years, younger than that of most published studies describing DRF in premenopausal women.) In premenopausal women, the circumstantial setting of fracture may involve the combined influence of greater body weight and height and of slightly narrower radius. That is, given greater height and BMI, there may be greater forward momentum and inertial force associated with a fall, and the narrower radius would confer a biomechanical disadvantage upon impact, with the two factors leading to fracture upon falling (even though bone status is categorized as normal). Furthermore, such fractures tend to be more severe, as patients with high BMI had a greater proportion of intraarticular fractures. 

Other investigators have concluded that smaller bones and high BMI influenced the risk for fracture. Skaggs, et al. reported that in a cohort of 50 Caucasian females aged 4-15 years (mean 9.6 years) with a forearm fracture, contralateral radii had on average an 8% smaller cross-sectional area (measured using computed tomography) compared to matched controls [11]. Trabecular and cortical BMD was not different between females with fractures and controls. The fracture group tended to be overweight. Skaggs, et al. concluded that the combination of smaller cross-sectional area associated with being overweight increases the vulnerability to fracture after a fall. Wapniarz, et al. found that women (n = 29, mean age 51 years) with Colles fracture had greater body weight and height than control women without fracture (mean age 48 years) [12]. Goulding, et al. reported that being overweight was a significant risk factor for forearm fracture in children and adolescents (n = 90, ages 5-19 years) [13-14]. Szulc, et al. reported that men with fractures (n = 74, mean age 66 years) had lower width of tubular bones (femoral neck and ulna, but not radius) compared to men with similar body weight and height [15]. Young military recruits, both men and women, with lower extremity stress fractures had lower weight-adjusted bone width compared to recruits who did not have fractures [16]. Melton, et al. reported that smaller cortical thickness contributes to the lower axial rigidity of radius in women with fractures [17]. It is interesting to note that several recent studies have suggested that overweight or obese status in younger women may be associated with increased risk of a variety of musculoskeletal injuries as a result of increased force upon falling [18-20].

There are limitations and strengths to this current study. The most notable limitation is that all measurements and data are from the contralateral radius, not the radius that actually fractured. However, side-to-side (left-right) differences in bone size and BMD are generally small and, hence, the contralateral radius was considered a surrogate for the bone status of the fractured radius. Construction of the matched control subsets was somewhat subjective, but the results would have been the same even if comparing to the unadjusted database (data not shown). The width of the ultradistal radius—the actual region where fractures occurred—was not reported because the measurement of ultradistal width was considered unreliable from DXA scans due to the varied shape of that region and lack of consistent landmarks for measurements. The assumption was made that width at the 33% ROI was reflective of bone size. However, DXA provides only a limited amount of information about bone size; namely, the 33% ROI width is only in one dimension, the anteroposterior view. A future study using more refined techniques to measure bone size is warranted. Although large side-to-side differences in BMD in women from the general population are not typically observed (as opposed to athletes or laborers who favor one side in their activities), it would have been preferable to have patients with fractures in their only dominant or nondominant side. That said, preliminary analyses (not shown) did not show significant differences in radii width between dominant and nondominant sites in the DRF groups. A strength of the study is that it reports homogeneous cohorts with respect to race, age, BMI, and lack of medication use that could affect bone.

## Conclusions

In conclusion, as a group, premenopausal Caucasian women less than 49 years of age had normal BMD but smaller radial width and greater body size compared to postmenopausal women over 50 years and matched controls without fractures. The circumstantial setting of fracture in these women may thus involve greater inertial force from a fall which impacts the narrower radius, compared to fractures in older women who fracture as a result of low BMD.
